# Socioeconomic Status and Postpartum Depression Risk After the *Dobbs v Jackson Women’s Health Organization *Decision, Based on State Trigger Laws

**DOI:** 10.1001/jamanetworkopen.2025.57337

**Published:** 2026-02-03

**Authors:** Onur Baser, Facundo Sepulveda, Yuanqing Lu, Amy Endrizal

**Affiliations:** 1 School of Public Health, City University of New York, New York, New York; 2 Department of Economics, Bogazici University, Istanbul, Türkiye; 3 Department of Economics, University of Santiago, Santiago, Chile; 4 Columbia Data Analytics, Ann Arbor, Michigan

## Abstract

**Importance:**

The 2022 Supreme Court decision in *Dobbs v Jackson Women’s Health Organization* resulted in immediate abortion bans or severe restrictions in 22 US states. While mental health consequences of restricted abortion access have been suggested, their distribution across socioeconomic strata remain unclear for postpartum depression (PPD) among Medicaid populations.

**Objective:**

To assess associations of state-level abortion bans enacted after *Dobbs* with the incidence of PPD, focusing on socioeconomic status (SES) differences.

**Design, Setting, and Participants:**

This retrospective cohort study used a difference-in-differences (DD) analysis for Medicaid claims data from Kythera Labs (approximately 60% of US Medicaid population) from January 2019 to December 2024. Women and adolescents aged 12 to 55 years with pregnancies resulting in live births or stillbirths were stratified into SES terciles based on zip code–level census data.

**Exposure:**

State-level abortion bans or restrictions implemented after *Dobbs*, defined by residence in trigger law states.

**Main Outcomes and Measures:**

The primary outcome was incidence of PPD within 12 months following delivery, identified through validated claims-based algorithms.

**Results:**

The study comprised 102 597 individuals pre-*Dobbs* (mean [SD] age, 27.21 [5.82] years; 47 305 individuals in trigger states and 55 292 individuals in nontrigger states) and 61 113 individuals post-*Dobbs* (mean [SD] age, 27.48 [5.92] years; 30 451 individuals in trigger states and 30 662 individuals in nontrigger states). Individuals in trigger states were younger than individuals in nontrigger states both pre-*Dobbs* (mean [SD] age, 26.53 [5.69] years vs 27.97 [5.84] years) and post-*Dobbs* (mean [SD] age, 26.61 [5.77] years vs 28.34 [5.94] years). Both pre- and post-*Dobbs*, individuals in trigger states were more likely to reside in rural areas (pre-*Dobbs*: 10 562 individuals [22.33%] vs 10 079 individuals [18.23%]; post-*Dobbs*: 6739 individuals [22.13%] vs 5220 individuals [17.02%]) and low-SES areas (pre-*Dobbs*: 20 136 individuals [42.57%] vs 13 771 individuals [24.91%]; post-*Dobbs*: 12 924 individuals [42.44%] vs 7283 [23.75%]); they were less likely to have obstetrical complications (pre-*Dobbs*: 31 243 individuals [66.05%] vs 42 780 individuals [77.37%]; post-*Dobbs*: 21 052 individuals [69.13%] vs 24 577 individuals [80.15%]) or maternal complications (pre-*Dobbs*:7732 individuals [16.34%] vs 10 839 of individuals [19.60%]; post-*Dobbs*: 5779 of 30 451 individuals [19.04%] vs 6508 individuals [21.17%]). Women with lowest SES residing in trigger states experienced a 9.0% relative increase in PPD diagnoses post-*Dobbs* vs similar women in nontrigger states (DD coefficient, 0.090; 95% CI, 0.035-0.146; *P* = .001). No associations were observed in middle or high SES groups.

**Conclusions and Relevance:**

In this cohort study, state-level abortion bans following *Dobbs* were associated with a disproportionate increase in the risk of PPD among women and adolescents in low-SES communities. These findings underscore the need for targeted mental health support and policy interventions to mitigate the unequal burden of such legislation on vulnerable populations.

## Key Points

**Question:**

Are state abortion bans enacted after the *Dobbs v Jackson Women’s Health Organization* decision associated with increased rates of postpartum depression (PPD) among Medicaid enrollees, based on socioeconomic status (SES)?

**Findings:**

This cohort study including 102 597 individuals pre-*Dobbs* and 61 113 individuals post-*Dobbs* found that women with low SES who lived in states with abortion bans experienced a significant 9.0% relative increase in PPD diagnoses after *Dobbs* vs those in states without bans. No differences in rates of PPD after *Dobbs* were observed among women in trigger vs nontrigger states living in middle- or high-SES areas.

**Meaning:**

This study found that post-*Dobbs* abortion bans were disproportionately associated with an increased PPD risk among individuals in low-SES communities, amplifying existing health disparities.

## Teaser

This cohort study analyzes associations of state-level abortion bans enacted after the *Dobbs v Jackson Women’s Health Organization *decision with incidence of postpartum depression among individuals with varying levels of socioeconomic status (SES).

## Introduction

When the US Supreme Court’s decision in *Dobbs v Jackson Women’s Health Organization*, 597 US 215 (June 24, 2022), reverted abortion regulation to the states, previously enacted trigger laws immediately went into effect in several states. Eventually, 14 states implemented near-total or total abortion bans, while 7 others established gestational limits ranging from 6 to 18 weeks.^1^

Restricted access to abortion after *Dobbs* has been associated with increased self-reported anxiety and depression symptoms.^2^ Similarly, Dave et al^3^ reported a 10% increase on a measure of mental distress in a population of female individuals of reproductive age. A recent analysis of survey data indicated that residence in states with trigger abortion bans, compared with residence in states without such bans, was associated with a modest yet statistically significant increase in symptoms of anxiety and depression following *Dobbs*.^1,4^ Research conducted prior to this decision also demonstrated that being denied an abortion was associated with adverse mental health outcomes.^2,5,6^ Most published studies, however, have assessed mental health in the general population of women of reproductive age, not specifically among pregnant women.

Even with unrestricted access to abortion services, the baseline risk for mental health disorders, and postpartum depression (PPD) in particular, varies systematically with income and other socioeconomic variables, with one study finding that low socioeconomic status (SES)^7^ is associated with PPD rates 11 times greater than high SES in first-time mothers.^8^ Taken together, these findings provide strong reasons to hypothesize that the associations of *Dobbs* with pregnant women’s mental health are heterogeneous by SES, with women from lower-SES communities experiencing larger increases in PPD rates.

This study used Medicaid claims data to examine the changes in PPD diagnoses associated with abortion restrictions after *Dobbs*. The sample was categorized into 3 SES groups based on zip code–level terciles, and a difference-in-differences (DD) model was used to estimate associations for each group in states that passed laws limiting abortion access.

## Methods

### Study Design and Data Source

This retrospective cohort study utilized Kythera Medicaid files from January 2019 to December 2024. The Kythera database is a comprehensive health care claims repository that encompasses medical and pharmacy claims data, providing coverage for approximately 79% of the US patient population.^9^ The dataset contains 60% of the Medicaid population across all 50 states and includes deidentified patient numbers, age, sex, insurance types (fee-for-service vs managed care), zip codes, and diagnoses according to the *International Classification of Diseases, Tenth Revision, Clinical Modification* (*ICD-10-CM*) codes.^10^ Each patient is assigned a unique identifier, which links all encounters and facilitates longitudinal analysis. The comprehensive details of the Kythera data have been documented in previous publications.^11,12,13^ Furthermore, the health care outcomes derived from these data have been validated and compared with other datasets for consistency and accuracy.^14^

Ethics approvals and informed consent for this study were not required because the data utilized were from an anonymous, deidentified database compliant with the Health Insurance Portability and Accountability Act (HIPAA). Institutional review board approval for research using a deidentified database is not required unless the researcher has access to a link allowing reidentification of data (45 CFR 46). Kythera Labs data have been determined by the Datavant Privacy Hub (Mirador) to comply with statistical deidentification required by HIPAA and associated regulations. Reporting follows the Strengthening the Reporting of Observational Studies in Epidemiology (STROBE) reporting guideline.^2,29^

### SES Score

The primary exposure was a zip code–level SES index summarizing neighborhood socioeconomic conditions, constructed from aggregated measures of income, housing value, investment income, educational attainment, and professional employment from the 2021 American Community Survey 5-year estimates, with standardized component *z* scores summed so that higher values indicate greater socioeconomic advantage.^15,16^ The 5-year survey period allows for more accurate estimation of characteristics that may have low prevalence or fluctuate over time, such as certain demographic traits or socioeconomic indicators in small geographic areas. We then linked this information to the enrollees’ zip code of residence in the Kythera files.

Previous research used factor analysis of census block groups to identify 6 variables that could be meaningfully combined into a summary SES score.^7^ These variables include 3 measures of wealth and income (log of the median household income,^16^ log of the median value of housing units,^17^ and the percentage of household receiving interest, dividend, or net rental income^18^), 2 measures of education (percentage of adults aged ≥25 years who completed high school or the percentage of adults aged ≥25 years who completed college),^15^ and 1 measure of occupation and employment (percentage of employed persons aged ≥16 years in executive, managerial, or professional specialty occupations).^15^ The *z *score for each variable is calculated by subtracting the overall mean and dividing it by the SD. For example, if the *z* score for zip code 10013 for the median housing income is 5, the median household income among the residents in that zip code is 5 SD higher than the overall median of housing income in the US. The SES score was then constructed by summing the *z *scores for each of the 6 variables.

Of the 33 774 zip code areas, 4912 had missing data, with 4161 having 3 or fewer missing variables. To address this, the method of missing data imputation with grouping or stratification was applied. Each variable was divided into 10 tiers based on dataset size, and missing data were filled using medians within each tier. After imputation, the data were combined with the existing population data, resulting in comprehensive information for 29 167 zip code areas. Summary SES scores ranged from −10.6320 to 23.0279, with larger scores corresponding to greater socioeconomic advantage. Participants were sorted according to summary SES scores and grouped in terciles. SES characteristics are shown in eTable 1 in Supplement 1.

### Analytic Data Set

Two distinct analytic samples were created for women in each of the 3 SES groups, corresponding to the periods before and after the June 2022 *Dobbs* decision. The study population comprised women and adolescents aged 12 to 55 years who experienced a pregnancy resulting in a live birth or stillbirth between January 2019 and December 2024 (study period). The pregnancy date was designated as the index date. Participants whose pregnancy concluded between January 2020 and June 2021 were assigned to the pre-*Dobbs* cohort, while those with pregnancy dates between June 2022 and December 2023 constituted the post-*Dobbs* cohort (eFigure 1 in Supplement 1). To ensure comprehensive assessment of comorbidities and of maternal, obstetrical, and lifestyle risk factors, a 12-month continuous enrollment period was required in the year prior to the index date. Similarly, a 12-month continuous enrollment period post–index date was mandated to capture PPD diagnoses. PPD was identified using the *ICD-10-CM* code F53x. The diagnostic criteria included at least 1 inpatient claim, or 1 outpatient claim for PPD, or a minimum of 1 inpatient or 2 outpatient claims for major depressive disorder occurring at least 27 days apart within 12 months following the end-of-pregnancy date or during the third trimester of pregnancy. To isolate PPD as a distinct condition, participants with a history of mania or bipolar disorder during the study period were excluded. Furthermore, to differentiate new-onset depressive illness in the postpartum period from ongoing episodes, patients with major depressive disorder prior to their last menstrual period were also excluded from the analysis. eFigure 2 in Supplement 1 summarizes the construction of the dataset. To define the exposure and comparison groups, we constructed a binary variable indicating residence in 1 of the 14 states with trigger laws or in 1 of the 36 nontrigger states across 2 distinct periods. Trigger states included Alabama, Arkansas, Idaho, Indiana, Kentucky, Louisiana, Mississippi, Missouri, Oklahoma, South Dakota, Tennessee, Texas, West Virginia, and North Dakota.

### Explanatory Variables

The demographic covariates included age, region, and urbanicity (urban, suburban, rural). Clinical factors encompassed comorbidity indexes, specifically the Charlson Comorbidity Index,^19^ Elixhauser Index,^20^ and Chronic Disease Score,^21^ as well as obstetrical, maternal, and lifestyle risk factors identified by *ICD-10-CM* codes (eTable 2 in Supplement 1). We also incorporated statewide variables that could potentially influence PPD diagnosis rates. These included the unemployment rate,^22^ education score, Substance Abuse and Mental Health Services Administration (SAMHSA) block grants for mental illness and substance use disorder prevention and treatment per capita,^23^ and the number of behavioral health care clinicians per 1000 state residents,^24^ obtained from the US Census Bureau County Business Patterns. Additionally, we accounted for the presence of Medicaid Health Home programs and Patient Protection and Affordable Care Act (ACA)–related Medicaid expansion in each state.

### Statistical Analysis

**Figure 1.**
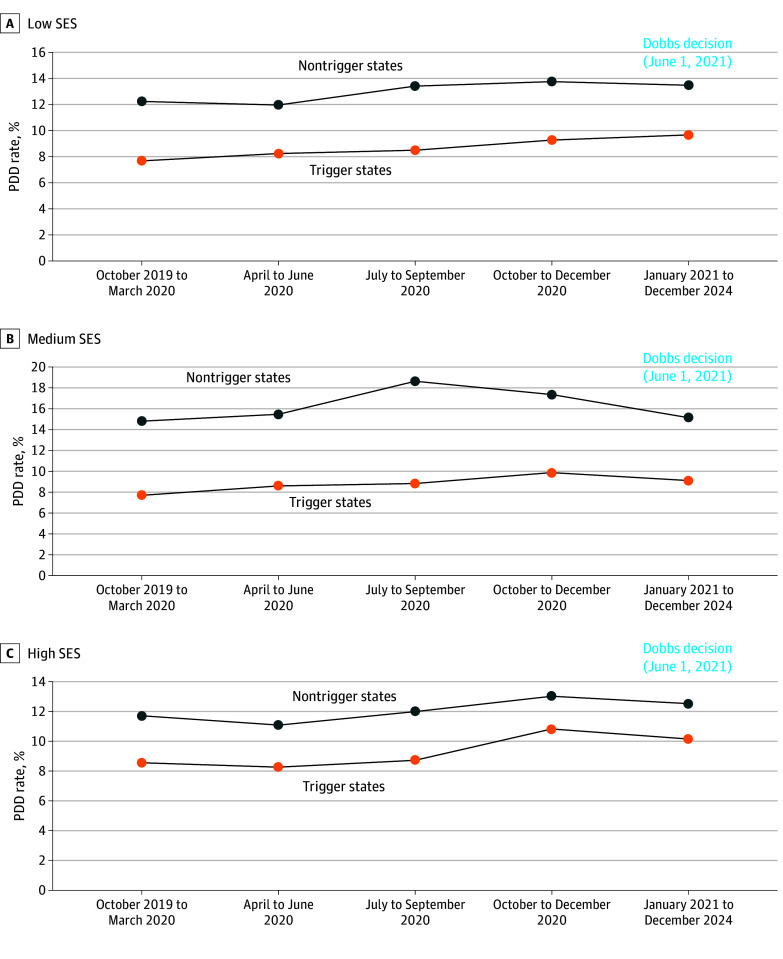
Parallel Trends in Postpartum Depression (PPD) Rates in Trigger vs Nontrigger States by Socioeconomic Status (SES)

A descriptive analysis of patients in each of the 3 SES areas was conducted, comparing those residing in states exposed to abortion bans (14 trigger states) with those in states without such bans (36 nontrigger states, including the District of Columbia), pre- and post-*Dobbs*. A DD approach was employed to estimate changes among residents of trigger states post-*Dobbs* relative to pre-*Dobbs* changes in nontrigger states. The DD method is widely used to assess the impact of policy interventions implemented at a specific time point.^25,26^ It compares changes over time between a group exposed to a policy and an unexposed group, attributing the DD to the policy’s effect. This approach assumes that both groups would have followed similar trends in the absence of the intervention, which is called the parallel trends assumption.^27^ To validate this assumption, we compared PPD rates between trigger and nontrigger states during the pre-*Dobbs* period. These trends are depicted in Figure 1. A quantitative assessment of differences in baseline periods and a statistical test of the visual parallel trends are reported in eTable 3 in Supplement 1.

Several strategies were implemented to address potential selection bias in this DD study. Demographic and clinical controls were included in the regression models to mitigate across-group bias arising from systematic differences between individuals in affected and unaffected states. To address across-time bias, the sample was restricted to individuals with continuous enrollment throughout the study period. Furthermore, standard errors were clustered by state to account for potential intrastate correlations because observations within the same state may not be independent.

Multiple model estimations were conducted to address Texas’s unique legislative context, analyzing data from all states, excluding Texas, and focusing solely on Texas. This approach accounted for the impact of Texas SB8, a law implemented in September 2021 that banned abortions after 6 weeks of gestation through civil penalties. This legislation led to a 50% reduction in abortion rates in Texas, marking a significant shift approximately 10 months before *Dobbs*.^28^

Analyses were conducted using R version 4.4.0 (R Project for Statistical Computing) within the Databricks analytics platform, with data processing performed using Pyspark. Results were considered statistically significant at a 2-sided *P* < .05.

## Results

After applying all the inclusion and exclusion criteria described previously, the final study sample included 102 597 individuals in the pre-*Dobbs* period and 61 113 individuals in the post-*Dobbs *period. During the pre-*Dobbs* period, 47 305 individuals resided in trigger states and 55 292 resided in nontrigger states, while during the post-*Dobbs* period, 30 451 individuals resided in trigger states and 30 662 individuals resided in nontrigger states (eFigure 2 in Supplement 1). Pre-*Dobbs*, the mean (SD) age of pregnant individuals in our sample was 27.21 (5.82) years. Pre-*Dobbs* 10 219 participants (9.96%) lived in urban areas, 71 767 participants (69.95%) lived in suburban areas, and the remainder lived in rural areas. Of these participants, 37 642 (36.68%) resided in states in the lowest tercile of behavioral health care clinicians per 1000 residents, 19 123 (18.64%) lived in states with SAMHSA mental health grant funding in the lowest tercile, 22 405 (21.84%) lived in states with a Medicaid Health Home for mental illness, and 76 577 (74.64%) lived in states with ACA Medicaid expansion. Of these participants, 74 023 (72.14%) had any obstetrical complications, 18 571 (18.10%) had any maternal comorbidity, and 18 141 (17.68%) had at least 1 lifestyle risk factor. Among pregnant individuals in the pre-*Dobbs* period, 11 201 (10.92%) received a PPD diagnosis. Post-*Dobbs*, these characteristics were similar; specifically, the mean (SD) was 27.48 (5.92) years, 6495 (10.63%) resided in urban areas, 42 660 (69.81%) lived in suburban areas, and the remainder lived in rural areas, with comparable distributions across behavioral health clinician supply, SAMHSA grant terciles, Medicaid Health Homes, ACA Medicaid expansion status, obstetrical complications, maternal comorbidities, and lifestyle risk factors.

In the pre-*Dobbs* period, there were 34 836 individuals in the high SES group, 33 854 in the medium SES group, and 34 017 in the low SES group. In the post-*Dobbs* period, there were 20 581 individuals in the high SES group, 20 342 individuals in the medium SES group, and 20 207 individuals in the low SES group. The demographic, socioeconomic, and clinical characteristics, as well as state-level factors for trigger and nontrigger states and by SES group, during the pre- and post-*Dobbs* periods are shown in the Table. No missing data were present in the final analytic dataset. Continuous enrollment in the Medicaid plan ensured that no participants were lost to follow-up during the study period.

**Table.** Patient Characteristics Before and After the *Dobbs *Decision, Stratified by Area SES and Trigger vs Nontrigger States

Patient characteristics	Pre-*Dobbs*			Post-*Dobbs*		
	Trigger states, No./total No. (%)	Nontrigger states, No./total No. (%)	STD	Trigger states, No./total No. (%)	Nontrigger states, No./total No. (%)	STD
Age						
Mean (SD), y						
All SES	26.53 (5.69)	27.97 (5.84)	0.25	26.61 (5.77)	28.34 (5.94)	0.29
Low SES	26.26 (5.68)	27.52 (5.83)	0.22	26.33 (5.78)	27.89 (5.97)	0.27
Medium SES	26.56 (5.66)	27.46 (5.70)	0.16	26.58 (5.68)	27.98 (5.87)	0.24
High SES	26.97 (5.73)	28.64 (5.89)	0.29	27.13 (5.82)	28.87 (5.94)	0.30
12-17 y						
All SES	973/47 305 (2.06)	701/55 292 (1.27)	0.06	672/30 451 (2.21)	361/30 662 (1.17)	0.08
Low SES	490/20 136 (4.17)	229/13 771 (1.66)	0.05	325/12 924 (2.51)	107/7283 (1.47)	0.07
Medium SES	273/15 611 (1.75)	241/18 243 (1.32)	0.03	200/9981 (2.00)	148/10 361 (1.43)	0.04
High SES	210/11 558 (1.82)	231/23 278 (0.99)	0.07	147/7546 (1.94)	106/13 035 (0.81)	0.10
≥18 y						
All SES	46 332/47 305 (97.94)	54 591/55 292 (98.73)	0.06	29 779/30 451 (97.79)	30 301/30 662 (98.83)	0.08
Low SES	19 646/20 136 (97.57)	13 542/13 771 (98.34)	0.05	12 599/12 924 (97.49)	7176/7283 (98.53)	0.07
Medium SES	15 338/15 611 (98.25)	18 002/18 243 (98.68)	0.03	9781/9981 (98.00)	10 213/10 361 (98.57)	0.04
High SES	11 348/11 558 (98.18)	23 047/23 278 (99.01)	0.07	7399/7546 (98.05)	12 929/13 035 (99.19)	0.10
Pandemic period indicator						
All SES	38 523/47 305 (81.43)	45 650/55 292 (82.56)	0.03	NA	NA	NA
Low SES	16 405/20 136 (81.47)	11 371/13 771 (82.57)	0.03	NA	NA	NA
Medium SES	12 766/15 611 (81.78)	14 991/18 243 (82.17)	0.01	NA	NA	NA
High SES	9352/11 558 (80.91)	19 288/23 278 (82.86)	0.05	NA	NA	NA
Region: South						
All SES	36 681/47 305 (77.54)	3335/55 292 (6.03)	2.15	24 346/30 451 (79.95)	2938/30 662 (9.58)	2.00
Low SES	16 631/20 136 (82.59)	552/13 771 (4.01)	2.48	11 009/12 924 (85.18)	546/7283 (7.50)	2.39
Medium SES	11 649/15 611 (74.62)	745/18 243 (4.08)	2.14	7714/9981 (77.29)	678/10 361 (6.54)	2.07
High SES	8401/11 558 (72.69)	2038/23 278 (8.76)	1.85	5623/7546 (74.52)	1714/13 035 (13.15)	1.63
Comorbidity score						
Charlson Comorbidity Index score, mean (SD)						
All SES	1.11 (0.38)	1.12 (0.39)	0.02	1.12 (0.41)	1.13 (0.40)	0.02
Low SES	1.11 (0.39)	1.13 (0.42)	0.06	1.14 (0.46)	1.12 (0.38)	0.03
Medium SES	1.10 (0.38)	1.11 (0.40)	0.01	1.11 (0.39)	1.13 (0.42)	0.06
High SES	1.11 (0.36)	1.11 (0.37)	0.01	1.11 (0.38)	1.13 (0.39)	0.04
Chronic Disease score, mean (SD)						
All SES	2.11 (1.42)	2.15 (1.45)	0.02	2.33 (1.55)	2.29 (1.55)	0.03
Low SES	2.10 (1.42)	2.13 (1.44)	0.02	2.33 (1.53)	2.25 (1.53)	0.05
Medium SES	2.14 (1.44)	2.18 (1.45)	0.03	2.35 (1.56)	2.35 (1.59)	9.18
High SES	2.10 (1.39)	2.12 (1.46)	0.02	2.33 (1.59)	2.25 (1.52)	0.06
Elixhauser Index score, mean (SD)						
All SES	1.60 (0.99)	1.71 (1.08)	0.11	1.69 (1.08)	1.83 (1.18)	0.12
Low SES	1.60 (1.00)	1.77 (1.14)	0.16	1.70 (1.12)	1.82 (1.17)	0.10
Medium SES	1.59 (0.98)	1.72 (1.08)	0.12	1.67 (1.07)	1.89 (1.22)	0.19
High SES	1.61 (0.98)	1.68 (1.04)	0.07	1.68 (1.05)	1.78 (1.14)	0.09
Location						
Urban						
All SES	5663/47 305 (11.91)	4556/55 292 (8.24)	0.12	3757/30 451 (12.34)	2738/30 662 (8.93)	0.11
Low SES	2673/20 136 (13.27)	554/13 771 (4.02)	0.32	1931/12 924 (14.94)	423/7283 (5.81)	0.29
Medium SES	1875/15 611 (12.01)	1756/18 243 (9.63)	0.08	1250/9981 (12.52)	1072/10 361 (10.35)	0.07
High SES	1085/11 558 (9.39)	2246/23 278 (9.65)	0.01	576/7546 (7.63)	1264/13 035 (9.56)	0.07
Suburban						
All SES	31 110/47 305 (65.76)	40 657/55 292 (73.53)	0.17	19 956/30 451 (65.53)	22 704/30 662 (74.05)	0.19
Low SES	10 450/20 136 (51.90)	10 924/13 771 (79.33)	0.59	6547/12 924 (50.66)	5644/7283 (77.50)	0.57
Medium SES	10 772/15 611 (69.00)	10 687/18 243 (58.58)	0.22	6885/9981 (68.98)	6442/10 361 (62.18)	0.14
High SES	9888/11 558 (85.56)	19 046/23 278 (81.82)	0.10	6524/7546 (86.46)	1163/13 035 (8.92)	0.11
Rural						
All SES	10 562/47 305 (22.33)	10 079/55 292 (18.23)	0.10	6739/30 451 (0.22)	5220/30 662 (0.17)	0.13
Low SES	7013/20 136 (34.83)	2293/13 771 (16.65)	0.41	4446/12 924 (34.40)	1216/7283 (16.70)	0.40
Medium SES	2964/15 611 (18.99)	5800/18 243 (31.79)	0.30	1846/9981 (18.50)	2847/10 361 (27.48)	0.21
High SES	585/11 558 (5.06)	1986/23 278 (8.53)	0.13	446/7546 (5.91)	1163/13 035 (8.92)	0.11
Behavioral health care clinicians, tercile						
1 (Low)						
All SES	32 527/47 305 (68.76)	5115/55 292 (9.25)	1.57	20 411/30 451 (67.03)	2719/30 662 (8.87)	1.50
Low SES	14 390/20 136 (71.46)	1062/13 771 (7.71)	1.65	9126/12 924 (70.61)	569/7283 (7.81)	1.58
Medium SES	10 454/15 611 (66.97)	2155/18 243 (11.81)	1.39	6664/9981 (66.77)	948/10 361 (9.15)	1.48
High SES	7683/11 558 (66.47)	1898/23 278 (8.15)	1.66	4621/7546 (61.24)	1202/13 035 (9.22)	1.39
2 (Middle)						
All SES	12 086/47 305 (25.55)	25 280/55 292 (45.72)	0.43	7702/30 451 (25.29)	15 977/30 662 (52.10)	0.57
Low SES	4942/20 136 (24.54)	6916/13 771 (50.22)	0.55	5743/12 924 (25.21)	3206/7283 (24.81)	0.71
Medium SES	4012/15 611 (25.70)	9573/18 243 (53.46)	0.59	2314/9981 (21.18)	5864/10 361 (56.60)	0.72
High SES	3132/11 558 (27.10)	8611/23 278 (36.99)	0.21	2182/7546 (28.92)	5946/13 035 (45.62)	0.35
3 (High)						
All SES	2692/47 305 (5.69)	24 897/55 292 (45.03)	0.99	2338/30 451 (7.68)	11 966/30 662 (39.03)	0.8
Low SES	804/20 136 (3.99)	5793/13 771 (42.07)	1.08	592/12 944 (4.58)	2530/7283 (34.74)	0.91
Medium SES	1145/15 611 (7.33)	6335/18 243 (34.72)	0.70	1003/9981 (10.05)	3549/10 361 (34.25)	0.61
High SES	743/11 558 (6.42)	12 769/23 278 (54.85)	1.12	743/7546 (9.85)	5887/13 035 (45.16)	0.81
SAMHSA grant funding tercile						
1 (Low)						
All SES	11 822/47 305 (24.99)	7302/55 292 (13.21)	0.31	8244/30 451 (27.07)	4243/30 662 (13.84)	0.33
Low SES	4760/20 136 (23.64)	1549/13 771 (11.25)	0.33	3318/12 924 (25.67)	828/7283 (11.37)	0.36
Medium SES	4125/15 611 (26.42)	2738/18 243 (15.00)	0.29	2858/9981 (28.64)	1450/10 361 (13.99)	0.36
High SES	2937/11 558 (25.41)	3015/23 278 (12.95)	0.33	2068/7546 (27.41)	1965/13 035 (15.07)	0.31
2 (Middle)						
All SES	8303/47 305 (17.55)	20 382/55 292 (36.86)	0.44	5642/30 451 (18.53)	13 639/30 662 (44.48)	0.58
Low SES	3383/20 136 (16.80)	4799/13 771 (34.85)	0.42	2338/12 924 (18.09)	2968/7283 (40.75)	0.53
Medium SES	3045/15 611 (19.50)	7694/18 243 (42.18)	0.50	1993/9981 (19.97)	5102/10 361 (49.24)	0.65
High SES	1875/11 558 (16.22)	7889/23 278 (33.89)	0.4	2182/7546 (28.92)	5946/13 035 (45.62)	0.35
3 (High)						
All SES	27 180/47 305 (57.46)	27 608/55 292 (49.93)	0.15	2338/30 451 (7.68)	11 966/30 662 (39.03)	0.80
Low SES	11 993/20 136 (59.56)	7423/13 771 (53.90)	0.12	7268/12 924 (56.24)	3470/7283 (47.65)	0.17
Medium SES	8441/15 611 (54.07)	7811/18 243 (42.81)	0.23	5130/9981 (51.40)	3809/10 361 (36.76)	0.30
High SES	6746/11 558 (58.37)	12 374/23 278 (53.16)	0.10	743/7546 (9.84)	5887/13 035 (45.16)	0.81
Medicaid Health Home for mental illness						
All SES	3730/47 305 (9.48)	18 675/55 292 (33.78)	0.60	1711/30 451 (6.57)	10 252/30 662 (33.43)	0.70
Low SES	1508/20 136 (9.03)	3960/13 771 (28.76)	0.53	740/12 924 (6.62)	2352/7283 (32.37)	0.73
Medium SES	1435/15 611 (10.77)	7350/18 243 (40.29)	0.70	683/9981 (7.94)	3743/10 361 (36.13)	0.91
High SES	787/11 558 (8.42)	9814/23 278 (42.16)	0.97	288/7546 (4.59)	4157/13 035 (31.89)	1.07
ACA Medicaid expansion						
All SES	23 622/47 305 (49.93)	52 955/55 292 (95.77)	1.23	15 189/30 451 (49.88)	29 144/30 662 (95.05)	1.17
Low SES	9093/20 136 (45.16)	13 292/13 771 (96.52)	1.24	5707/12 924 (44.16)	6963/7283 (95.61)	1.24
Medium SES	8316/15 611 (53.27)	17 055/18 243 (93.49)	1.05	5143/9981 (51.53)	9667/10 361 (93.40)	1.07
High SES	6213/11 558 (53.75)	22 608/23 278 (97.12)	1.36	4339/7546 (57.50)	12 504/13 035 (95.93)	1.14
Any obstetrical complications						
All SES	31 243/47 305 (66.05)	42 780/55 292 (77.37)	0.25	21 052/30 451 (69.13)	24 577/30 662 (80.15)	0.26
Low SES	13 258/20 136 (65.84)	10 655/13 771 (77.37)	0.25	9064/12 924 (70.13)	5727/7283 (78.64)	0.19
Medium SES	10 165/15 611 (65.11)	14 191/18 243 (77.79)	0.28	6679/9981 (66.92)	8424/10 361 (81.30)	0.33
High SES	7820/11 558 (67.66)	17 934/23 278 (77.04)	0.21	5309/7546 (70.36)	10 439/13 035 (80.08)	0.23
Any maternal comorbidity						
All SES	7732/47 305 (16.34)	10 839/55 292 (19.60)	0.08	5799/30 451 (1904)	6508/30 662 (21.22)	0.05
Low SES	3383/20 136 (16.80)	2715/13 771 (19.72)	0.07	2523/12 924 (19.52)	1542/7283 (21.17)	0.04
Medium SES	2450/15 611 (15.70)	3615/18 243 (19.82)	0.11	1857/9981 (18.61)	2251/10 361 (21.73)	0.08
High SES	1899/11 558 (16.43)	4509/23 278 (19.37)	0.07	1419/7546 (0.19)	2719/13 035 (20.86)	0.05
Lifestyle risk factors						
All SES	6420/47 305 (13.57)	11 721/55 292 (21.20)	0.20	5283/30 451 (17.35)	7987/30 662 (26.05)	0.21
Low SES	2691/20 136 (13.36)	2945/13 771 (21.39)	0.21	2291/12 924 (17.73)	1927/7283 (26.46)	0.22
Medium SES	2005/15 611 (12.84)	3950/18 243 (21.65)	0.23	1675/9981 (16.78)	2844/10 361 (27.45)	0.26
High SES	1724/11 558 (14.92)	4826/23 278 (20.73)	0.15	1317/7546 (17.45)	3223/13 035 (24.73)	0.18
PPD diagnosis among pregnant women						
All SES	4025/47 305 (13.57)	6996/55 292 (12.65)	0.13	2892/30 451 (9.50)	4148/30 662 (13.53)	0.13
Low SES	1580/20 136 (7.85)	1556/13 771 (11.30)	0.12	1193/12 924 (9.23)	814/7283 (11.18)	0.07
Medium SES	1354/15 611 (8.67)	2670/18 243 (14.63)	0.19	928/9981 (9.30)	1642/10 361 (15.85)	0.2
High SES	1091/11 558 (9.44)	2770/23 278 (11.90)	0.08	771/7546 (10.22)	1693/13 035 (12.99)	0.08

Abbreviations: ACA, Patient Protection and Affordable Care Act; *Dobbs, Dobbs v Jackson Women’s Health Organization*; NA, not applicable; PPD, postpartum depression; SAMHSA, Substance Abuse and Mental Health Services Administration; SES, socioeconomic status; STD, standardized difference.

Analysis revealed significant differences between individuals in trigger and nontrigger states, which appeared mostly consistent across SES groups. Residents of trigger states were younger (pre-*Dobbs*: mean [SD] age, 26.53 [5.69] years vs 27.97 [5.84] years; post-*Dobbs* (mean [SD] age, 26.61 [5.77] years vs 28.34 [5.94] years), more often located in the South (pre-*Dobbs*: 36 681 individuals [77.54%] vs 3335 individuals [6.03%]; post-*Dobbs*: 24 346 individuals [79.95%] vs 2938 individuals [9.58%]), more often located in rural areas (pre-*Dobbs*: 10 562 individuals [22.33%] vs 10 079 individuals [18.23%]; post-*Dobbs*: 6739 individuals [22.13%] vs 5220 individuals [17.02%]), had a higher rate of adolescent pregnancy (pre-*Dobbs*: 973 individuals [2.06%] vs. 701 individuals [1.27%]; post-*Dobbs*: 672 individuals [2.21%] vs. 361 individuals [1.17%]) and faced greater socioeconomic challenges (pre-*Dobbs*: 20 136 individuals [42.57%] vs 13 771 individuals [24.91%]; post-*Dobbs*: 12 924 individuals [42.44%] vs 7283 [23.75%]). Despite these socioeconomic disadvantages, residents of trigger states had lower rates of maternal comorbidities (pre-*Dobbs*: 7732 individuals [16.34%] vs 10 839 of individuals [19.60%]; post-*Dobbs*: 5779 individuals [19.04%] vs 6508 individuals [21.17%]), lifestyle risk factors (pre-*Dobbs*: 6420 individuals [13.57%] vs 11 721 individuals [21.20%]; post-*Dobbs*: 5283 individuals [17.35%] vs 7987 individuals [26.05%]), and obstetrical complications (pre-*Dobbs*: 31 243 individuals [66.05%] vs 42 780 individuals [77.37%]; post-*Dobbs*: 21 052 individuals [69.13%] vs 24 577 individuals [80.15%]) compared with those in nontrigger states. Compared with nontrigger states, patients in trigger states had fewer behavioral health care clinicians per 1000 residents, received less per-capita funding from SAMHSA block grants for mental illness and substance use disorder prevention and treatment, and were less likely to have implemented Medicaid Health Home programs or expanded Medicaid under the ACA (Table).

**Figure 2.**
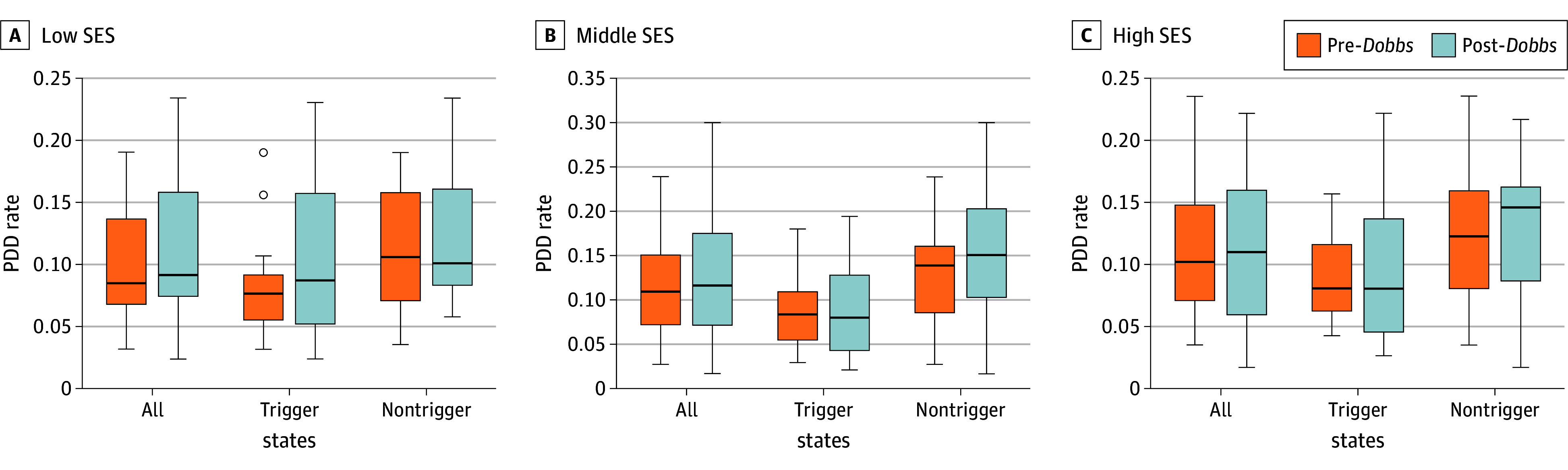
Variation in Postpartum Depression (PPD) Rates Before and After the *Dobbs v Jackson Women’s Health Organization *Decision in Low, Middle, and High Socioeconomic Status (SES) Groups The line within the box represents the median, with the upper and lower edges of the box reflecting the IQR and the bars reflecting the range. Circles indicate outliers.

Detailed obstetrical and maternal complications and lifestyle factors can be found in eTable 4 in Supplement 1. Variation in PPD rates between the pre- and post-*Dobbs* periods became more pronounced in trigger states than in nontrigger states (Figure 2). The rate change for each state is presented in eFigure 3 and eFigure 4 in Supplement 1.

**Figure 3.**
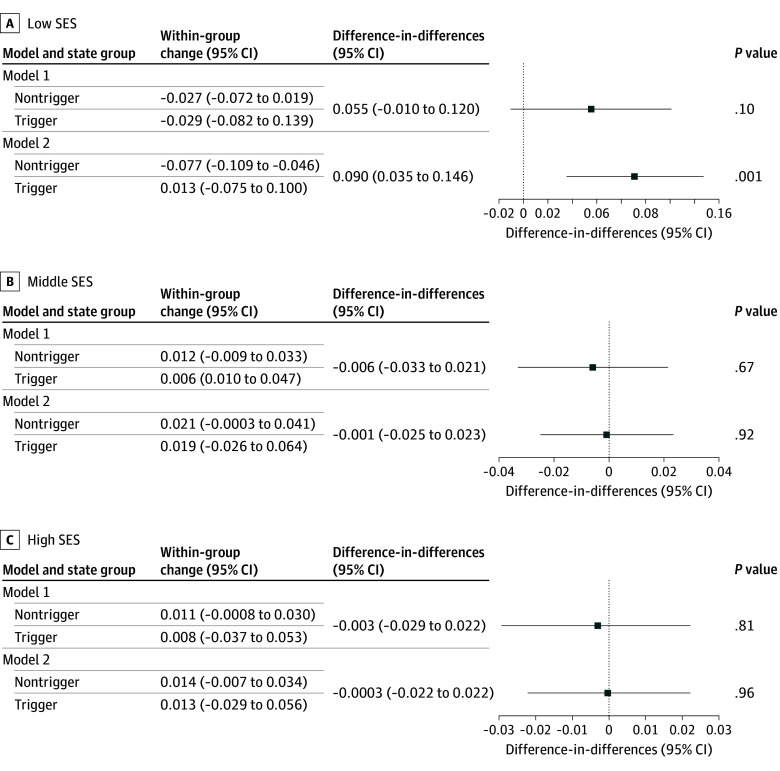
Difference-in-Difference Models Comparing Postpartum Depression (PPD) Diagnosis Rates in Low, Middle, and High Socioeconomic Status (SES) Groups Before and After the *Dobbs v Jackson Women’s Health Organization *Decision in Trigger vs Nontrigger States Within-group change represents the difference in PPD diagnosis rates post-*Dobbs* vs pre-*Dobbs* in both trigger and nontrigger states. The difference-in-differences column was obtained by subtracting the nontrigger estimate from the trigger estimate in the previous column and constitutes our main set of results. By construction, all coefficients represent proportional changes. In particular, difference-in-differences coefficients represent the proportional change in PPD rates associated with the passing of trigger legislation, while accounting for time trends in PPD rates.

DD analysis results are presented in Figure 3 for all 3 groups. The DD analysis included 2 models. The first—model 1—did not adjusted for covariates. The second—model 2—adjusted for most of the covariates listed in Table, which we expect helps mitigate selection bias. For Low-SES areas (Figure 3A), our preferred estimate, in model 2, shows a positive and statistically significant DD coefficient of 0.090 (95% CI, 0.035 to 0.146), indicating that residing in a trigger state was associated with a 9% increase in the incidence of a PPD diagnosis. In this case, compared with the pre-*Dobbs* period, within-group changes results show that PPD diagnosis rates did not significantly increase in trigger states (within-group change, 0.013; 95% CI, −0.075 to 0.100), but decreased significantly by 7.7% in nontrigger states after *Dobbs *(within-group change, −0.077; 95% CI, −0.035 to −0.146). For model 1, the magnitude of the DD estimate was 5.5% (0.055; 95% CI, −0.010 to 0.020) but was insignificant.

For medium SES (Figure 3B) and high SES (Figure 3C), the coefficients were close to 0 for both models and were smaller in absolute value by at least 1 order of magnitude relative to those with low SES. These coefficients were statistically insignificant at standard significance levels.

Given the particular restrictions on abortion implemented by Texas before *Dobbs*, a sensitivity analysis was conducted to assert the importance of this jurisdiction for our results. This analysis involved 2 key steps. Texas was excluded from the sample, and the regressions were rerun, yielding results similar to the overall sample trends. Second, we conducted separate regression analyses using only data from Texas, which revealed that PPD diagnosis rates were significantly higher in Texas following *Dobbs* (eTable 5 in Supplement 1).

## Discussion

Our findings in this cohort study revealed heterogeneous associations of *Dobbs* with PPD rates across SES groups. For the groups with higher SES scores, no associations were found. For the lowest SES group, living in a state that restricted access to abortion after *Dobbs* was associated with a 9.0% (95% CI, 3.5%-14.5%) increase in PPD rates (*P* = .001).

Women living in trigger states do have options when dealing with an unwanted pregnancy, but these options are financially costly, which drives a wedge between the outcomes of women in lower vs medium to high SES communities. The most common option is traveling to a state where abortion is legal.^30,31,32^ One estimate showed that under the current access landscape, approximately 75% of pregnant residents seeking access from banned states will travel to facilities in states without such bans.^30^ This estimate is supported by current reports indicating increased abortion rates in states with legal abortion adjacent to states with bans.^33^ This alternative is costly. First, the distance to the nearest abortion facility increased from an average of 43 miles one-way before *Dobbs* to 330 miles at present. Second, Medicaid only covers in-state care in most states.

A second option to end an unwanted pregnancy in the context of a state that limits access to abortion is the use of abortion-inducing medications. Rise in demand for programs like Aid Access, a nonprofit organization that provides paid mail-order access to medication abortion, for a fee, confirms that individuals unable to manage the logistics and costs for traveling for the service, may opt for self-managed abortions without substantial health risk.^34^

Differences in the availability of support programs and mental health infrastructure between states with abortion bans (trigger states) and those without (nontrigger states) may have systematically biased the estimated impacts of *Dobbs* on PPD rates in the Medicaid population studied. Residents of trigger states, regardless of SES, had markedly less access to core mental health support programs, including SAMHSA block grants (lower per capita funding), fewer Medicaid Health Home programs for mental illness, less widespread ACA Medicaid expansion, and notably fewer behavioral health care clinicians per 1000 people compared with nontrigger states. These gaps were most pronounced for individuals residing in low SES areas, who, as the analysis shows, were more exposed to restrictive abortion policy and less able to benefit from the protective effects of available mental health services. Lower availability of these programs in trigger states may have inflated the measured association of abortion bans with PPD risk, because part of the observed increase in PPD among individuals with low SES could stem from a relative lack of mental health infrastructure and programmatic supports, independent of abortion legislation itself. Underinvestment in SAMHSA block grants, Medicaid Health Homes, and fewer behavioral health clinicians all contribute to increased barriers in accessing preventive and treatment services for depression and related mental health disorders. Similarly, states that did not expand Medicaid under the ACA further limited access to behavioral health for economically vulnerable populations. This study’s DD approach adjusted for these state-level characteristics as covariates in multivariate models but cannot fully rule out residual confounding.

### Limitations

This study has limitations. Our findings may have limited generalizability because the analysis is restricted to Medicaid-covered deliveries, excluding commercially insured births, which account for roughly 60% of deliveries in the US. Medicaid enrollees are typically from lower SES communities than privately insured individuals, and the Kythera data lack information on race, both of which are important determinants of PPD risk.

Although models adjust for observable characteristics such as age, comorbidities, SES, and state-level differences, unmeasured factors (eg, genetics, social support, and major life events) may still confound the association of the *Dobbs* decision with PPD risk if they correlate with policy exposure. In addition, the data do not allow for identification of patients who traveled from trigger-law states to nontrigger states for abortion care. As a result, the forced births captured in our sample likely represent individuals with particularly limited resources to travel or access medication abortion, which may introduce selection bias; however, the relative socioeconomic homogeneity of the Medicaid population may attenuate this concern. To further address potential travel-related bias, a sensitivity analysis excluding beneficiaries from trigger states that border nontrigger states (Alabama and Georgia) yielded results consistent with the main findings.

The study excluded individuals who obtained abortion services and does not distinguish whether the pregnancies and births in the sample were intended or wanted, which may be important determinants of PPD risk. As with all administrative claims data, this analysis is subject to potential misclassification, coding errors, and the absence of detailed clinical and patient-reported information. Because PPD is underdiagnosed, particularly among disadvantaged groups, our findings pertain only to diagnosed PPD and may underestimate the true burden of disease.

## Conclusions

This cohort study contributes to the ongoing discourse on the mental health implications of abortion legislation. Based on this analysis of Medicaid data spanning December 2019 to December 2024, statistically significant differences in PPD diagnosis rates were found between trigger and nontrigger states following *Dobbs* among women and adolescents who resided in lower SES areas. These findings underscore the need for targeted mental health support and policy interventions to mitigate the unequal burden of restrictive reproductive legislation on vulnerable populations.

## Notes

Supplement 1. eTable 1. Characteristics of Medicaid-Enrolled Patients According to Summary SES Score Tercile by US Zip Code eFigure 1. Study Design and Timeline Pre- and Post-Dobbs eFigure 2. Attrition Table for Study Sample eTable 2. *ICD-10* Codes eTable 3. Parallel Trends Test for the Difference-in-Differences Model by SES Group eTable 4. Obstetrical Complications, Comorbidities, and Lifestyle Risk Factors Among Women in Trigger vs Non-Trigger States, Pre- and Post-Dobbs eFigure 3. PPD Rate Trends in Trigger States Among Low-SES Groups eFigure 4. PPD Rate Trends in Non-trigger States Among Low-SES Groups eTable 5. Regression Model Predicting PPD Rates, Texas Only

Supplement 2. Data Sharing Statement
