# Supplementary material for: Socioeconomic Status and Postpartum Depression Risk After the Dobbs v Jackson Women’s Health Organization Decision, Based on State Trigger Laws
Source: JAMA Netw Open. 2026 Feb 3;9(2):e2557337. doi: 10.1001/jamanetworkopen.2025.57337 (PMC12869335; doi:10.1001/jamanetworkopen.2025.57337)
Supplement: Supplement 1. — eTable 1. Characteristics of Medicaid-Enrolled Patients According to Summary SES Score Tercile by US Zip Code eFigure 1. Study Design and Timeline Pre- and Post-Dobbs eFigure 2. Attrition Table for Study Sample eTable 2. ICD-10 Codes eTable 3. Parallel Trends Test for the Difference-in-Differences Model by SES Group eTable 4. Obstetrical Complications, Comorbidities, and Lifestyle Risk Factors Among Women in Trigger vs Non-Trigger States, Pre- and Post-Dobbs eFigure 3. PPD Rate Trends in Trigger States Among Low-SES Groups eFigure 4. PPD Rate Trends in Non-trigger States Among Low-SES Groups eTable 5. Regression Model Predicting PPD Rates, Texas Only [file jamanetwopen-e2557337-s001.pdf]

Supplemental Online Content

Baser O, Sepulveda F, Lu Y, Endrizal A. Socioeconomic status and postpartum depression risk after the *Dobbs v. Jackson Women's Health Organization* decision based on state trigger laws. *JAMA Network Open*. 2025;9(2):e2557337. doi:10.1001/jamanetworkopen.2025.57337

eTable 1. Characteristics of Medicaid-Enrolled Patients According to Summary SES Score Tercile by US Zip Code

eFigure 1. Study Design and Timeline Pre- and Post-*Dobbs*

eFigure 2. Attrition Table for Study Sample

eTable 2. *ICD-10* Codes

eTable 3. Parallel Trends Test for the Difference-in-Differences Model by SES Group

eTable 4. Obstetrical Complications, Comorbidities, and Lifestyle Risk Factors Among Women in Trigger vs Non-Trigger States, Pre- and Post-*Dobbs*

eFigure 3. PPD Rate Trends in Trigger States Among Low-SES Groups

eFigure 4. PPD Rate Trends in Non-trigger States Among Low-SES Groups

eTable 5. Regression Model Predicting PPD Rates, Texas Only

This supplemental material has been provided by the authors to give readers additional information about their work.

© 2026 Baser O et al. *JAMA Network Open*.

eTable 1. Characteristics of Medicaid-Enrolled Patients According to Summary SES Score Tercile by US Zip Code

	**Socioeconomic Area**		
	**1 (Low)**	**2 (Middle)**	**3 (High)**
No. of patients	54,224	53,038	54,404
Median SES summary score (range)	-0.1609<br>(-9.0778, 1.777)	3.0488<br>(1.7421, 4.6176)	6.7410<br>(4.5769, 23.0279)
Wealth/income			
Median household income ($)	45,457	60,047	85,714
Median value of housing unit ($)	125,700	176,600	347,500
Household with interest, dividend, or rental income (%)	10.54	17.33	26.79
Education			
Adult residents who completed at least high school (%)	79.11	89.63	94.23
Adult residents who completed at least college (%)	16.59	27.20	47.48
Employment			
Employed residents with management, professional, and related occupations (%)	25.94	35.22	50.51

© 2026 Baser O et al. *JAMA Network Open*.

eFigure 1. Study Design and Timeline Pre- and Post-*Dobbs*

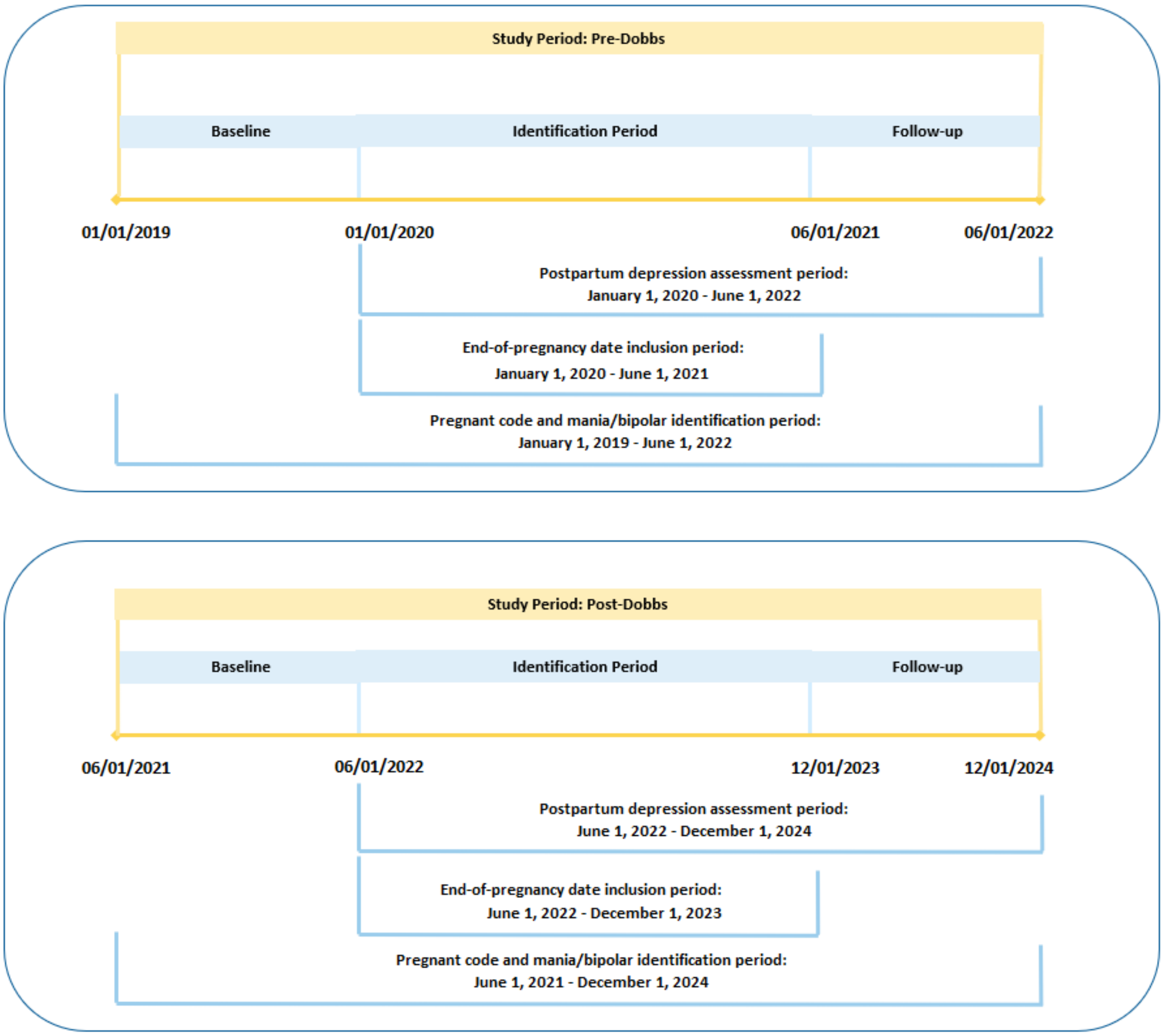

© 2026 Baser O et al. *JAMA Network Open*.

eFigure 2. Attrition Table for Study Sample

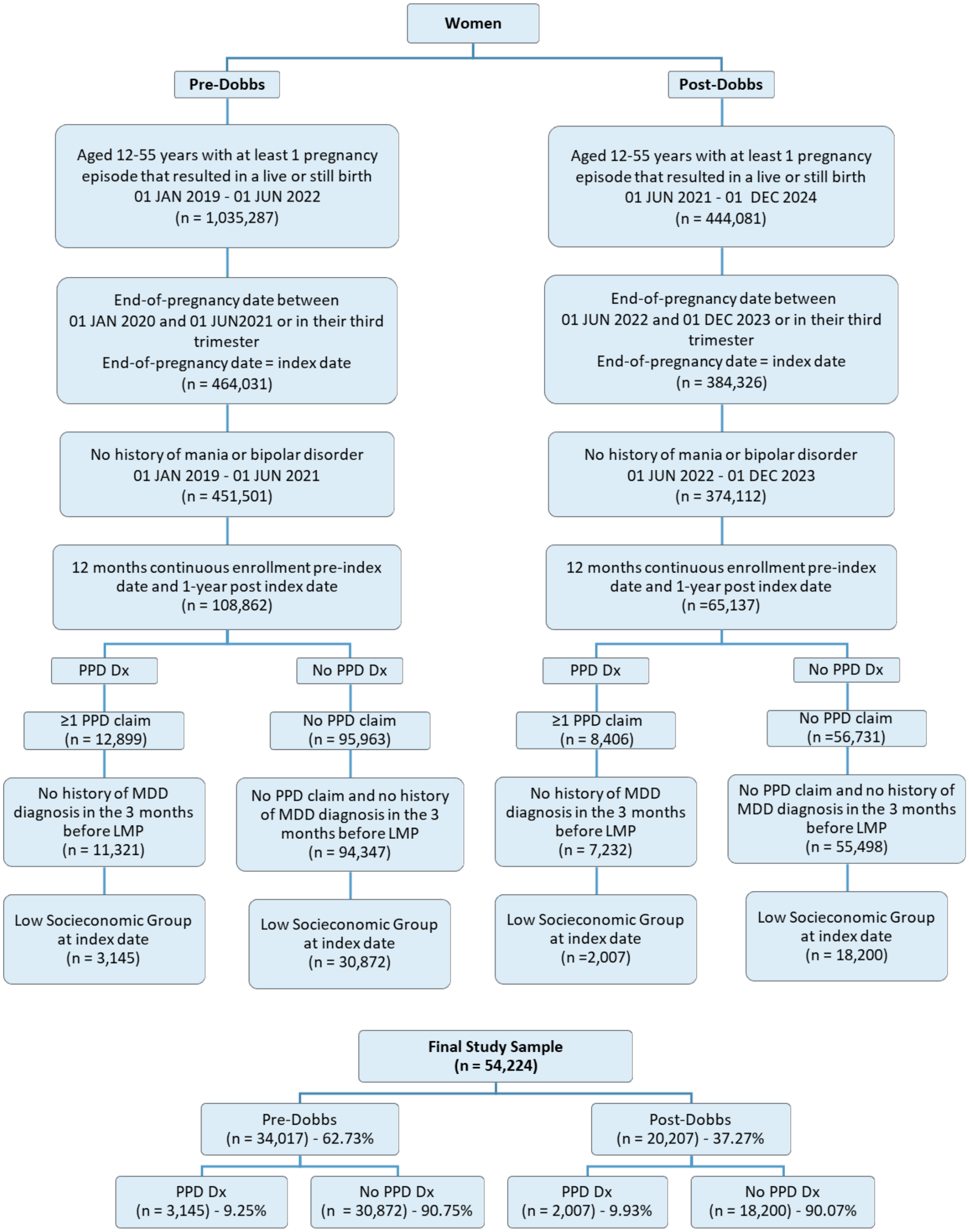

Abbreviations: Dx, diagnosis; LMP, last missed period; MDD, major depressive disorder; PPD, postpartum depression.

© 2026 Baser O et al. *JAMA Network Open*.

eTable 2. *ICD-10* Codes

**Condition**	***ICD-10* Code(s)**
Obstetrical complications	
Drug dependence	F19.20
High-risk pregnancy	O09.90, O09.899, O09.70
Severe perineal laceration	O703, O704
Postpartum hemorrhage	O72
Puerperal sepsis	O850
Infection of obstetric surgical wound	O860
Venous complication in puerperium	O870, O871, O873, O878, O879
Obstetric embolism	O88
Complication of anesthesia	O89
Complication of the puerperium	O90
Other maternal diseases	O98, O99
Cesarean section	O82
Multiple births	Z37.5, Z37.6, Z37.7
Preterm labor	O60
Abnormal findings	O28, O35, O36
Postpartum anemia	O90.81
Negative birth experience	Z87.59
Meconium passage	P03
Umbilical cord prolapse	O69.0
Prior history of abortion	Z33.2, N96, O02, O03*, O04*, O05*, O06*, O07*
Prior history of ectopic pregnancy	O09.10
Prior history of hydatidiform mole	O01.9
Other obstetric trauma	O71
Premature rupture of membranes	O42
Placental disorders	O43
Placenta previa	O44
Premature separation of the placenta	O45
Maternal comorbidities	

© 2026 Baser O et al. *JAMA Network Open*.

Condition	***ICD-10* Code(s)**
Preexisting hypertension	I10 obese
Gestational hypertension	O13.3
Pre-eclampsia or eclampsia	O11, O13, O14, O15, O16
Gestational diabetes mellitus	O24
Pre-existing diabetes	E11.9
Lifestyle risk factors	
Vitamin D deficiency	E55.9
Obese and overweight	E66
Sleep disorders	G47
Lack of physical exercise	Z72.3
Poor eating habits	Z72.4
Vitamin B6 deficiency	E53.1
Smoking	O99.330

Abbreviation: ICD-10, *International Classification of Diseases, Tenth Revision.*

© 2026 Baser O et al. *JAMA Network Open*.

eTable 3. Parallel Trends Test for the Difference-in-Differences Model by SES Group

Wave	Estimate	Standard Error	*P* Value
Low SES (A)			
2020/04–2020/06	-0.0009	0.007	0.90
2020/07–2020/09	-0.0001	0.007	0.99
2020/10–2020/12	0.009	0.006	0.18
Middle SES (B)			
2020/04–2020/06	0.002	0.008	0.84
2020/07–2020/09	-0.0003	0.007	0.96
2020/10–2020/12	0.003	0.007	0.70
High SES (C)			
2020/04–2020/06	0.008	0.007	0.30
2020/07–2020/09	0.008	0.007	0.25
2020/10–2020/12	0.011	0.007	0.11

Abbreviation: SES, socioeconomic status.

© 2026 Baser O et al. *JAMA Network Open*.

eTable 4. Obstetrical Complications, Comorbidities, and Lifestyle Risk Factors Among Women in Trigger vs Non-Trigger States, Pre- and Post-*Dobbs*

	**Pre-*Dobbs* (N=34,017)**					**Post-*Dobbs* (N = 20,207)**				
	**Trigger States (N=20,136)**		**Non-Trigger States (N=13,881)**		**Std. Diff.**	**Trigger States (N=12,924)**		**Non-Trigger States (N=7,283)**		**Std. Diff.**
	**N/Mean**	**%/SD**	**N/Mean**	**%/SD**		**N/Mean**	**%/SD**	**N/Mean**	**%/SD**	
Obstetrical complications										
Drug dependence	21	0.10%	36	0.26%	0.0379	15	0.12%	10	0.14%	0.0060
High-risk pregnancy	947	4.70%	1,239	9.00%	0.1733	833	6.45%	825	11.33%	0.1785
Severe perineal laceration	2	0.01%	0	0.00%	0.0130	3	0.02%	0	0.00%	0.0191
Postpartum hemorrhage	40	0.20%	27	0.20%	0.0009	34	0.26%	20	0.27%	0.0022
Puerperal sepsis	0	0.00%	0	0.00%		0	0.00%	0	0.00%	
Infection of obstetric surgical wound	4	0.02%	1	0.01%	0.0104	0	0.00%	1	0.01%	0.0195
Venous complication in puerperium	2	0.01%	1	0.01%	0.0029	0	0.00%	1	0.01%	0.0195
Obstetric embolism	18	0.09%	30	0.22%	0.0338	15	0.12%	19	0.26%	0.0353
Complication of anesthesia	1	0.00%	1	0.01%	0.0029	2	0.02%	1	0.01%	0.0014
Complication of the puerperium	81	0.40%	70	0.51%	0.0174	59	0.46%	35	0.48%	0.0035
Other maternal diseases	10,239	50.85%	8,564	62.19%	0.2270	7,334	56.75%	4,897	67.24%	0.2158
Cesarean section	62	0.31%	56	0.41%	0.0162	32	0.25%	23	0.32%	0.0131
Multiple births	1	0.00%	0	0.00%	0.0092	1	0.01%	0	0.00%	0.0110
Preterm labor	1,325	6.58%	886	6.43%	0.0068	776	6.00%	381	5.23%	0.0333
Abnormal findings	6,633	32.94%	5,876	42.67%	0.2022	4,822	37.31%	3,147	43.21%	0.1209
Postpartum anemia	37	0.18%	25	0.18%	0.0025	23	0.18%	16	0.22%	0.0095

© 2026 Baser O et al. *JAMA Network Open*.

	Pre-*Dobbs* (N=34,017)					Post-*Dobbs* (N = 20,207)				
	Trigger States (N=20,136)		Non-Trigger States (N=13,881)		Std. Diff.	Trigger States (N=12,924)		Non-Trigger States (N=7,283)		Std. Diff.
	N/Mean	%/SD	N/Mean	%/SD		N/Mean	%/SD	N/Mean	%/SD	
Negative birth experience	808	4.01%	1,038	7.54%	0.1542	811	6.28%	809	11.11%	0.1786
Meconium passage	3	0.01%	6	0.04%	0.0174	4	0.03%	2	0.03%	0.0020
Umbilical cord prolapse	4	0.02%	1	0.01%	0.0104	1	0.01%	4	0.05%	0.0300
Prior history of abortion	664	3.30%	633	4.60%	0.0684	512	3.96%	339	4.65%	0.0345
Prior history of ectopic pregnancy	7	0.03%	14	0.10%	0.0266	4	0.03%	6	0.08%	0.0231
Prior history of hydatidiform mole	4	0.02%	2	0.01%	0.0041	0	0.00%	0	0.00%	
Other obstetric trauma	89	0.44%	67	0.49%	0.0071	76	0.59%	45	0.62%	0.0039
Premature rupture of membranes	431	2.14%	306	2.22%	0.0049	292	2.26%	149	2.05%	0.0146
Placental disorders	325	1.61%	405	2.94%	0.0905	305	2.36%	239	3.28%	0.0570
Placenta previa	568	2.82%	560	4.07%	0.0693	377	2.92%	277	3.80%	0.0501
Premature separation of the placenta	54	0.27%	60	0.44%	0.0284	26	0.20%	22	0.30%	0.0207
Any obstetrical complications	13,258	65.84%	10,655	77.37%	0.2532	9,064	70.13%	5,727	78.64%	0.1928
Maternal comorbidities										
Preexisting hypertension	748	3.71%	568	4.12%	0.0203	605	4.68%	380	5.22%	0.0249
Gestational hypertension	727	3.61%	508	3.69%	0.0042	539	4.17%	284	3.90%	0.0137
Pre-eclampsia or eclampsia	1,837	9.12%	1,337	9.71%	0.0195	1,335	10.33%	744	10.22%	0.0038
Gestational diabetes mellitus	1,538	7.64%	1,431	10.39%	0.0955	1,144	8.85%	805	11.05%	0.0746
Pre-existing diabetes	318	1.58%	299	2.17%	0.0446	272	2.10%	167	2.29%	0.0129
Any maternal comorbidity	3,383	16.80%	2,715	19.72%	0.0742	2,523	19.52%	1,542	21.17%	0.0412

© 2026 Baser O et al. *JAMA Network Open*.

	Pre-*Dobbs* (N=34,017)					Post-*Dobbs* (N = 20,207)				
	Trigger States (N=20,136)		Non-Trigger States (N=13,881)		Std. Diff.	Trigger States (N=12,924)		Non-Trigger States (N=7,283)		Std. Diff.
	N/Mean	%/SD	N/Mean	%/SD		N/Mean	%/SD	N/Mean	%/SD	
Lifestyle risk factors										
Vitamin D deficiency	380	1.89%	319	2.32%	0.0299	438	3.39%	316	4.34%	0.0501
Obese and overweight	2,196	10.91%	2,513	18.25%	0.2116	1,821	14.09%	1,644	22.57%	0.2264
Sleep disorders	221	1.10%	218	1.58%	0.0425	180	1.39%	132	1.81%	0.0340
Lack of physical exercise	0	0.00%	4	0.03%	0.0266	4	0.03%	2	0.03%	0.0020
Poor eating habits	3	0.01%	3	0.02%	0.0051	1	0.01%	3	0.04%	0.0238
Vitamin B6 deficiency	0	0.00%	1	0.01%	0.0133	0	0.00%	0	0.00%	
Smoking	135	0.67%	168	1.22%	0.0575	87	0.67%	54	0.74%	0.0082
Any lifestyle risk factors	2,691	13.36%	2,945	21.39%	0.2147	2,291	17.73%	1,927	26.46%	0.2160

Abbreviation: Std. Diff., standardized difference.

© 2026 Baser O et al. *JAMA Network Open*.

eFigure 3. PPD Rate Trends in Trigger States Among Low-SES Groups

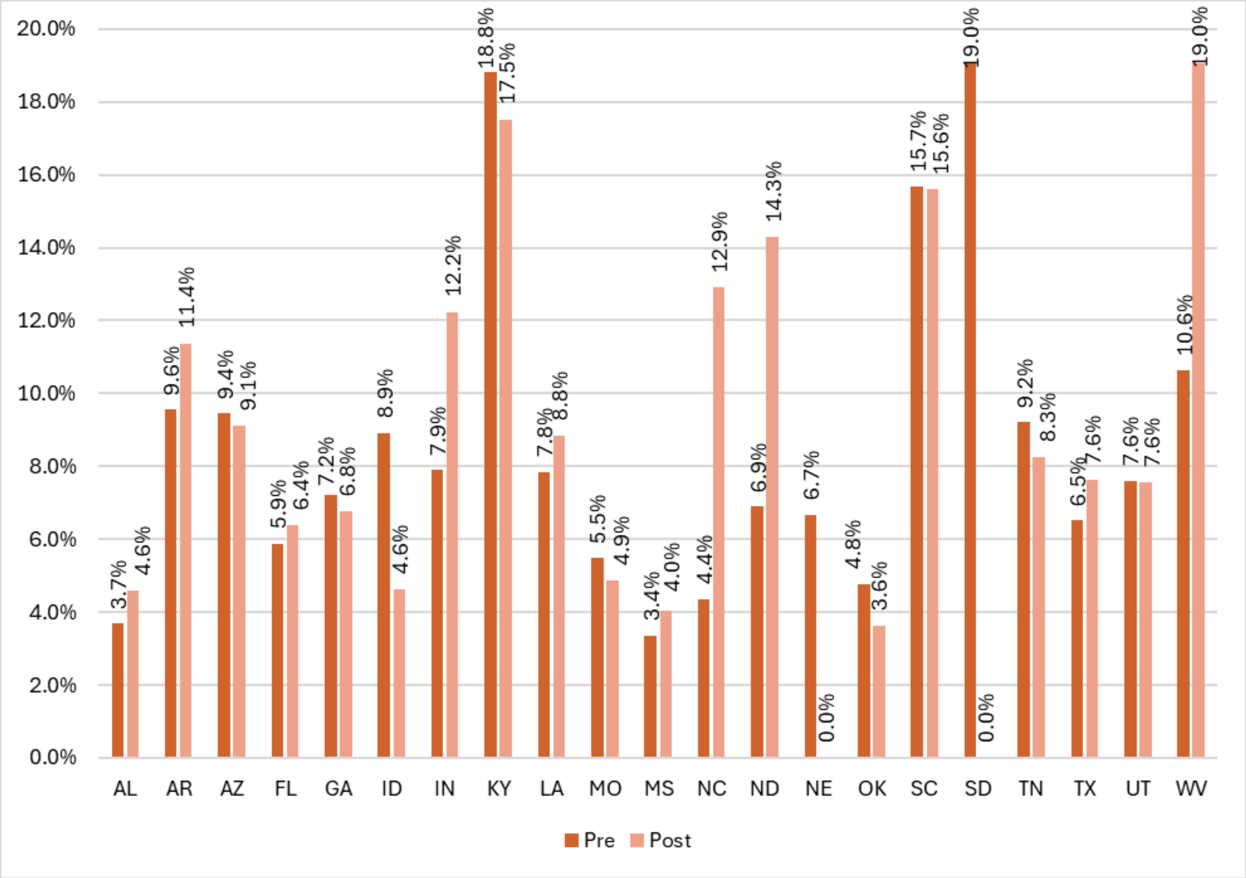

Abbreviations: PPD, postpartum depression; SES, socioeconomic status.

© 2026 Baser O et al. *JAMA Network Open*.

eFigure 4. PPD Rate Trends in Non-trigger States Among Low-SES Groups

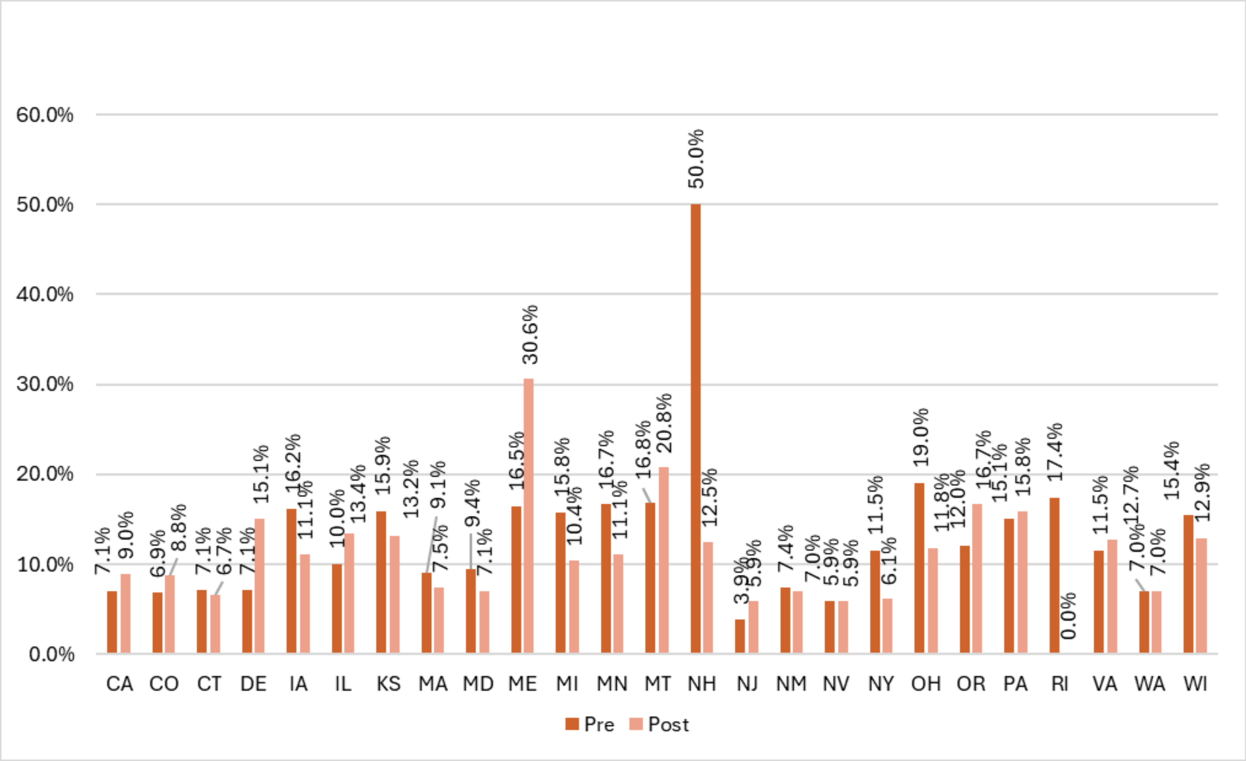

Abbreviations: PPD, postpartum depression; SES, socioeconomic status.

© 2026 Baser O et al. *JAMA Network Open*.

eTable 5. Regression Model Predicting PPD Rates, Texas Only

	**Women Aged 12-55 y**	
	**Unadjusted**	**Adjusted**
Post-*Dobbs*	0.0112*	0.9330
	(-0.0017,0.02401)	(0.0741,0.1067)
Constant	0.0651***	0.1180***
	(0.0571,0.0731)	(0.1029,0.1331)
Observations	6,321	6,318
Adjusted R^2	0.0003	0.0055
Residual std. error	0.2542	0.2535
F statistic	2.9*	9.8***

Abbreviation: PPD, postpartum depression.

**P* < .10.

*** *P* < .001.

© 2026 Baser O et al. *JAMA Network Open*.
