# Supplementary material for: Socioeconomic Status and Postpartum Depression Risk After the Dobbs v Jackson Women’s Health Organization Decision, Based on State Trigger Laws
Source: JAMA Netw Open. 2026 Feb 3;9(2):e2557337. doi: 10.1001/jamanetworkopen.2025.57337 (PMC12869335; doi:10.1001/jamanetworkopen.2025.57337)
Supplement: Supplement 2. — Data Sharing Statement [file jamanetwopen-e2557337-s002.pdf]

Data Sharing Statement

Baser. Socioeconomic Status and Postpartum Depression Risk After the Dobbs v Jackson Women's Health Organization Decision, Based on State Trigger Laws. *JAMA Netw Open*. Published February 03, 2026. doi:10.1001/jamanetworkopen.2025.57337

Data
Data available: No

Additional Information
Explanation for why data not available: Data used under license and may be made available from author Onur Baser upon reasonable request and permission of Kythera Labs.
